# Abnormal sodium and water homeostasis in mice with defective heparan sulfate polymerization

**DOI:** 10.1371/journal.pone.0220333

**Published:** 2019-07-31

**Authors:** Rik H. G. Olde Engberink, Judith de Vos, Angela van Weert, Yahua Zhang, Naomi van Vlies, Bert-Jan H. van den Born, Jens M. Titze, Ed van Bavel, Liffert Vogt

**Affiliations:** 1 Department of Internal Medicine, section Nephrology, Amsterdam UMC, University of Amsterdam, Amsterdam Cardiovascular Sciences, Amsterdam, The Netherlands; 2 Department of Biomedical Engineering and Physics, Amsterdam UMC, University of Amsterdam, Amsterdam Cardiovascular Sciences, Amsterdam, the Netherlands; 3 Department of Clinical Pharmacology, Vanderbilt University, Nashville, Tennessee, United States of America; 4 Laboratory of Genetic Metabolic Disease, Amsterdam UMC, University of Amsterdam, Amsterdam Cardiovascular Sciences, Amsterdam University Medical Centre, Amsterdam, the Netherlands; 5 Department of Internal Medicine, section Vascular Medicine, Amsterdam UMC, University of Amsterdam, Amsterdam Cardiovascular Sciences, Amsterdam, The Netherlands; University Medical Center Utrecht, NETHERLANDS

## Abstract

Glycosaminoglycans in the skin interstitium and endothelial surface layer have been shown to be involved in local sodium accumulation without commensurate water retention. Dysfunction of heparan sulfate glycosaminoglycans may therefore disrupt sodium and water homeostasis. In this study, we investigated the effects of combined heterozygous loss of heparan sulfate polymerization genes (exostosin glycosyltransferase 1 and 2; Ext1^+/-^Ext2^+/-^) on sodium and water homeostasis. Sodium storage capacity was decreased in Ext1^+/-^Ext2^+/-^ mice as reflected by a 77% reduction in endothelial surface layer thickness and a lower skin sodium-to-glycosaminoglycan ratio. Also, these mice were characterized by a higher heart rate, increased fluid intake, increased plasma osmolality and a decreased skin water and sodium content, suggesting volume depletion. Upon chronic high sodium intake, the initial volume depletion was restored but no blood pressure increase was observed. Acute hypertonic saline infusion resulted in a distinct blood pressure response: we observed a significant 15% decrease in control mice whereas blood pressure did not change in Ext1^+/-^Ext2^+/-^ mice. This differential blood pressure response may be explained by the reduced capacity for sodium storage and/or the impaired vasodilation response, as measured by wire myography, which was observed in Ext1^+/-^Ext2^+/-^ mice. Together, these data demonstrate that defective heparan sulfate glycosaminoglycan synthesis leads to abnormal sodium and water homeostasis and an abnormal response to sodium loading, most likely caused by inadequate capacity for local sodium storage.

## Introduction

Traditionally, the kidney is believed to be responsible for matching sodium excretion with sodium intake, thereby preventing sodium retention. However, long-term sodium balance studies have demonstrated that total body sodium content shows large fluctuations during fixed sodium intake. Surprisingly, these fluctuations did not result in changes of extracellular volume or body weight.[1, 2] As extracellular osmolality is tightly regulated, such variation in total body sodium without volume effects can only be explained by local sodium accumulation that is not accompanied by commensurate water retention. Subsequent studies demonstrated that sodium can accumulate in the endothelial surface layer (ESL) and the skin interstitium, where sodium is osmotically inactivated by negatively-charged glycosaminoglycans (GAGs) and does not induces water retention.[3–10] Moreover, recent data suggest that sodium is also actively concentrated in the skin interstitium.[11, 12] Local sodium storage may have a significant impact on sodium balance and osmoregulation. In healthy subjects, for example, 60 mmol of sodium was lost or appeared within four hours after hypertonic saline infusion or water loading, respectively.[13, 14] Also, skin sodium content has been shown to increase significantly after high salt intake.[7] Inadequate capacity for local sodium storage may therefore affect sodium and water homeostasis and result in an abnormal response to sodium excess.

Heparan sulfate is a GAG that is found both in the skin and ESL. In the skin, heparan sulfate is present in high concentrations in the dermo-epidermal junction while the dermis itself contains low amounts of heparan sulfate.[15, 16] Within the ESL, heparan sulfate is the predominant GAG accounting for 50–90% of total ESL GAG content.[17] We therefore hypothesized that defective heparan sulfate GAGs would decrease local sodium storage capacity and thereby affect sodium and water homeostasis. Heparan sulfate polymerization is regulated by exostosin glycosyltransferase 1 and 2 (Ext1 and Ext2) genes. Both, heterozygous loss of Ext1 or Ext2 has been shown to reduce heparan sulfate synthesis and ESL volume in mice.[18–20] To test whether heparan sulfate GAGs affect sodium and water homeostasis, we have investigated volume regulation and osmoregulation in mice with combined heterozygous loss of Ext1 and Ext2.

## Materials and methods

### Ethical approval

All experimental protocols were approved by the Animal Ethics Committee of the Academic Medical Centre, Amsterdam, the Netherlands (protocol number DIN102932). We adhered to the National Institute of Health (NIH) guide for the care and use of laboratory animals and institutional guidelines. All surgery was performed under anesthesia, and all efforts were made to minimize suffering. Mice were sacrificed by exsanguination from cardiac puncture under general anesthesia.

### Animals and sodium interventions

Single heterozygous Ext1 and Ext2 mice were fully backcrossed into a C57/BL6J background. We crossed Ext1^+/-^ and Ext2^+/-^ mice and used male, combined heterozygous Ext1 and Ext2 (Ext1^+/-^Ext2^+/-^) mice to ensure maximally impaired heparan sulfate GAG polymerization. Male C57/BL6J mice (control; Harlan Laboratories Inc.) were used for control experiments. All 84 mice that were used for this study were housed in a temperature controlled room with a 12:12 light-dark cycle.

We investigated the effects of a normal sodium diet as well as an acute and chronic sodium load. The chronic sodium load consisted of a one-week high sodium (8.0% NaCl) diet under ad libitum water drinking conditions. Control mice remained on the normal diet (0.3% NaCl). Acute sodium loading involved infusion of 1.8% NaCl (8 μl/g body weight) through the jugular vein in approximately 100 seconds.

### Plasma measurements

Blood samples for measurement of electrolytes were collected from the saphenous vein after each diet and were immediately analysed using the point of care I-STAT System and CG8+ cartridges (Abbot Point of Care Inc, Princeton, NJ, USA). Plasma osmolality was measured in a different sample using a freezing point depression osmometer.

### Blood pressure measurements

We performed non-invasive tail cuff blood pressure (BP) measurements in conscious mice using the volume pressure recording CODA system (Kent Scientific Corporation, CT, USA). To reduce stress and minimize variation in BP, we trained all mice for one week prior to the experiments. After at least 5 minutes of rest, we performed 15 measurements. Tail blood volume had to be at least 25 μl for acceptance of these data. All BP measurements were obtained between 15.00 and 18.00 pm. Investigators were not blinded to the genetic origin of the mice during BP measurements.

To investigate the effects of an acute sodium load, we measured intra-arterial BP. All mice were anesthetised with an intraperitoneal injection of a mixture (0.075 mL/10 gr) of ketamine (100 mg/ml), dexmedetomidine (0.5 mg/ml) and atropine (1 mg/ml). Foot reflexes were regularly checked to monitor anesthesia. During surgery and measurements, all mice were placed on a heating pad and body temperature was monitored. After induction of anesthesia, we inserted a PE-10 cannula in the carotid artery for continuous monitoring of arterial pressure and heart rate. We first performed baseline measurements when BP and heart rate were stable. During infusion we determined BP and heart rate at 5-second intervals. After infusion, we determined mean BP and heart rate at every minute for 10 minutes.

### Skin measurements

We used snap frozen abdominal skin samples to analyse skin water and sodium content as described previously.[21] In short, to determine skin water content we compared skin sample weights before and after desiccation at 90°C for 48 hours. Next, samples were dry ashed for 40 hours at 450°C. After ashing, all samples were dissolved in 5% HNO3. We used flame photometry to measure sodium and potassium concentrations.

To test the consequences of heterozygous loss of Ext1 and Ext2 on skin sodium storage capacity, we used high performance liquid chromatograph-mass spectrometry/mass spectrometry (HPLC-MS/MS) to measure heparan sulfate and dermatan sulfate disaccharides as previously described.[22, 23] In short, snap frozen skin samples were homogenised and protein content was determined.[24] GAGs in the homogenate were enzymatically digested and the disaccharides were quantified on a Waters Quattro Premier XE (tandem) mass spectrometer coupled to an Acquity UPLC system (UPLC-MS/MS). The disaccharides were separated on a Thermo Hypercarb HPLC column (100 × 2.1 mm, 5 μm). All disaccharides were detected and quantified in the MRM acquisition mode. All samples were analysed in duplicate. Disaccharide concentrations were normalized for homogenate protein content. If we were not able to detect certain disaccharides, we used the level of detection divided by two for analysis.

To assess whether defective heparan sulfate polymerization affects skin sodium storage we calculated skin sodium/heparan sulfate ratio. We presumed that a lower skin sodium/heparan sulfate ratio reflected a reduced capacity of heparan sulfate mediated skin sodium storage.

### Intravital microscopy

We determined ESL thickness with intravital microscopy considering that ESL thickness reflects heparan sulfate GAG content. We prepared the jugular vein for infusion of 40-kDa (Tetramethylrhodamine (TRITC-Dx40), excitation/emission 555/580 nm, Molecular Probes) and 500 kDa dextran solutions (Fluorescein isothiocyanate (FITC-Dx500), excitation/emission 490/520 nm, Sigma-Aldrich) (0.05 mL, 10 mg/mL in PBS). Subsequently, we prepared and pinned the cremaster muscle on a transparent silicon pedestal as previously described.[25] During surgery and measurements, the cremaster muscle was superperfused with a physiological salt solution at 37°C. For intravital imaging of the cremaster microvasculature, we used a Zeiss upright microscope mounted on an own-built stage equipped with an x60 water immersion objective (numerical aperture of 0.90; LUMPlanFl, Olympus). The cremaster muscle was epi-illuminated with a mercury lamp while using the appropriate filters for imaging of FITC-Dx500 and TRITC-Dx40 labelled dextran columns. After infusion of both dextrans, we randomly selected 15–20 vessels with a diameter of maximal 40 μm. A digital camera (Retiga-SRV Fast 1394, QImaging) was used to capture 5 images of both FITC-Dx500 and TRITC-Dx40 dextran columns of each vessel (field of view 164x123 μm (resolution 1380x1040)).

The widths of FITC-Dx500 and TRITC-Dx40 columns were measured by a blinded observer who drew three rectangles perpendicular to the longitudinal axis of the vessel. Along these rectangles, we measured the fluorescence intensity using ImageJ software. We calculated the inflection points on both sides of the sigmoidal shaped fluorescence intensity curve to determine the edges of the fluorescent column.[26] Next, we calculated the ESL thickness by subtracting the width of the FITC-Dx500 column, which does not include the ESL, from the TRITC-Dx40 column, which represents the total vessel diameter including the ESL (S1 Fig).[26]

### Wire myography

To assess whether BP may be affected by changes in endothelial function that may be anticipated following ESL modulation, we isolated second- or third-order mesenteric arteries (300–350 μm) for wire myography. Arterial segments of 2 mm were mounted into a multichannel wire myograph (610M, Danish Myo Technology, Aarhus, Denmark) for isometric tension measurements as previously described.[27] We first performed two contractions using a high potassium salt solution with a 30-minute washout in between. Then, after initial vasoconstriction with a thromboxane analogue U46119 (0.3 μM), we studied vasorelaxation at increasing methacholine concentrations (10 nmol/L—10 μmol/L).

### Statistics

Data are presented as mean and standard error of the mean (SEM). Baseline characteristics were compared using non-parametric tests or t-tests as appropriate. Longitudinal data and methacholine dose response curves were compared using a general linear model for repeated measurements with Bonferroni correction for multiple comparisons. Data were analysed using SPSS (Version 21.0, SPSS, Inc., Chicago, IL).

## Results

### Local sodium storage capacity is altered in Ext1^+/-^Ext2^+/-^ mice

**Fig 1:**
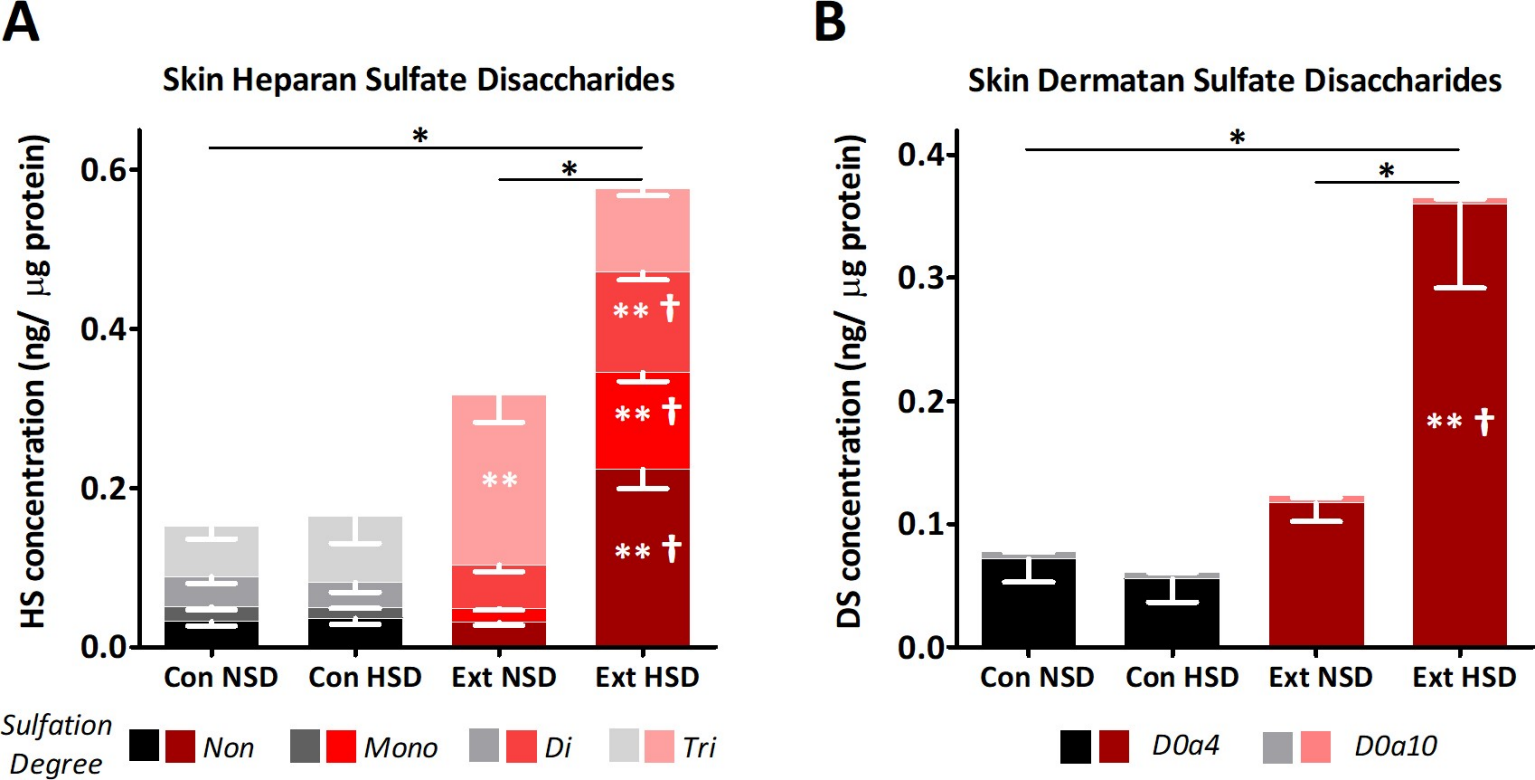
Altered skin GAG content and sulfation in Ext1^+/-^Ext2^+/-^ mice. Results from HPLC-MS/MS measurements of skin heparan sulfate (A) and dermatan sulfate (B) content and sulfation in Ext1^+/-^Ext2^+/-^ and control mice. (A) No difference in total skin heparan sulfate content was seen between Ext1^+/-^Ext2^+/-^ mice and controls on a NSD, but tri-sulfated heparan sulfate disaccharides were more prevalent in the skin of Ext1^+/-^Ext2^+/-^ mice. After a HSD, total skin heparan sulfate disaccharide content increased in Ext1^+/-^Ext2^+/-^ mice (p = 0.004) while no effect was seen in controls (p = 1.00; n = 5–6 for each condition). (B) Similar results were found for dermatan sulfate disaccharides (n = 5–6 for each condition): total skin dermatan sulfate disaccharide content was similar in Ext1^+/-^Ext2^+/-^ mice and controls (p = 0.25) and increased after HSD in Ext1^+/-^Ext2^+/-^ mice (p = 0.002), but not in controls (p = 0.84). *p<0.05; **compared to control mice on a similar diet; †compared to Ext1^+/-^Ext2^+/-^ on a NSD. Con, controls; DS, dermatan sulfate; Ext, Ext1^+/-^Ext2^+/-^; HS, heparan sulfate; HSD, high sodium diet; NSD, normal sodium diet.

**Fig 2:**
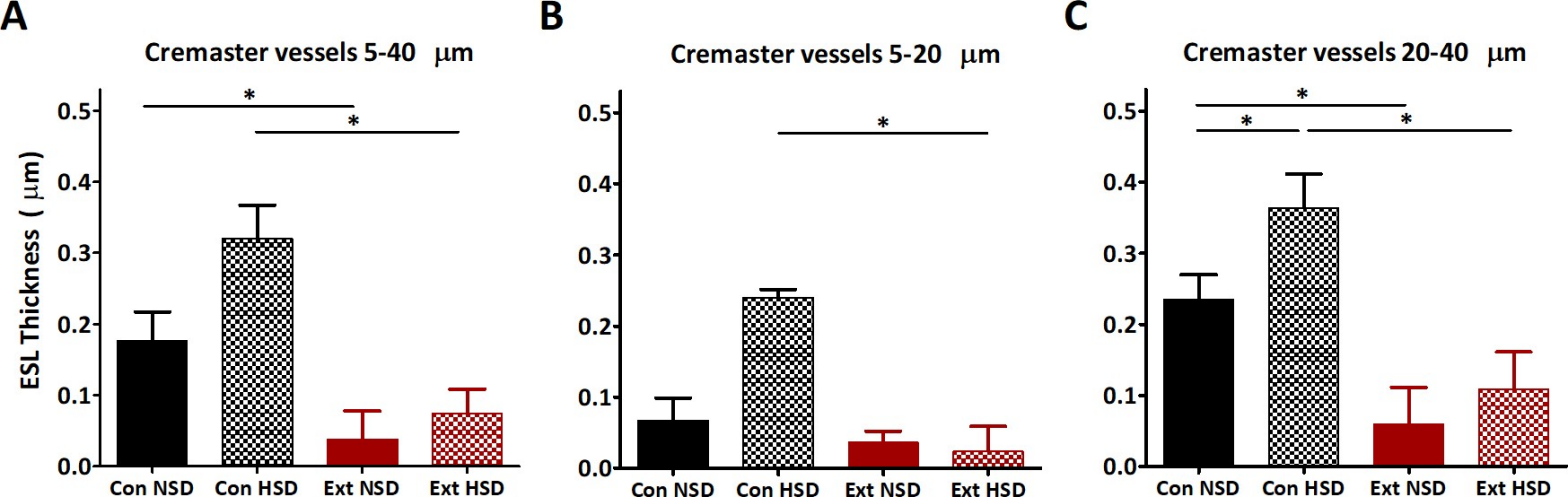
ESL thickness is reduced in Ext1^+/-^Ext2^+/-^ mice and increased by high sodium diet. (A) Control mice had a larger ESL (NSD: 0.18±0.04 μm, n = 6; HSD: 0.32±0.05 μm, n = 3) than Ext1^+/-^Ext2^+/-^ mice on both diets (NSD: 0.04±0.04 μm, n = 6, p = 0.04; HSD 0.07±0.03μm, n = 7, p = 0.004). There was no difference in ESL thickness between diets within control mice (p = 0.07) or Ext1^+/-^Ext2^+/-^ mice (p = 0.47). (B) In a subset of 5–20 μm vessels with a mean diameter of 12.7±0.8 μm, control (0.07±0.03μm, n = 4) and Ext1^+/-^Ext2^+/-^ mice (0.04±0.02 μm, n = 5) had equal ESL thickness on a NSD (p = 0.38). On a HSD, control mice (0.24±0.01 μm, n = 3) had a thicker ESL than Ext1^+/-^Ext2^+/-^ mice (0.02±0.04 μm, n = 7, p = 0.005). ESL thickness was not affected by diet within control mice (p = 0.06) or Ext1^+/-^Ext2^+/-^ mice (p = 1.00). (C) In 20–40 μm vessels with a mean diameter of 28.6±0.4 μm, controls (NSD: 0.24±0.03 μm, n = 6; HSD 0.36±0.05 μm, n = 3) had a significant larger ESL than Ext1^+/-^Ext2^+/-^ mice on both diets (NSD: 0.04±0.02 μm, n = 6, p = 0.02; HSD: 0.11±0.05μm, n = 7, p = 0.02). HSD increased ESL thickness in control mice (p = 0.02), but not in Ext1^+/-^Ext2^+/-^ mice (p = 0.53). *p<0.05. Con, controls; ESL, endothelial surface layer; Ext, Ext1^+/-^Ext2^+/-^; HSD, high sodium diet; NSD, normal sodium diet.

We investigated two potential compartments for sodium storage in Ext1^+/-^Ext2^+/-^ mice. First, we analysed skin GAG content. On a normal diet, skin heparan and dermatan sulfate disaccharide content were similar in Ext1^+/-^Ext2^+/-^ and control mice. However, significantly higher heparan sulfate sulfation degrees were found in Ext1^+/-^Ext2^+/-^ mice (Fig 1). Skin sodium/heparan sulfate disaccharide ratio was 65% lower in Ext1^+/-^Ext2^+/-^ mice compared to controls (2.1±0.3 vs. 0.7±0.2 mmol sodium/ng heparan sulfate; p = 0.009), which indicates impaired sodium storage capacity of skin heparan sulfates of Ext1^+/-^Ext2^+/-^ mice. Secondly, we assessed the ESL thickness. Intravital microscopy demonstrated that ESL thickness, in microvessels ranging from 5–40 μm, of Ext1^+/-^Ext2^+/-^ mice was significantly reduced with 77% (Fig 2A). The mean diameter of microvessels that were included in the analysis was 22.8±0.7 μm and did not differ among groups (p = 0.43). Because the effects of sodium may differ in vessels of different size, we separately analysed our data for the smallest (5–20 μm) and larger vessels (20–40 μm) of the microcirculation (Fig 2B and 2C). In vessels ranging from 5–20 μm, ESL thickness of control and Ext1^+/-^Ext2^+/-^ mice was not different. In 20–40 μm vessels, controls had a thicker ESL than Ext1^+/-^Ext2^+/-^ mice.

### Ext1^+/-^Ext2^+/-^ mice display abnormal volume regulation

**Fig 3:**
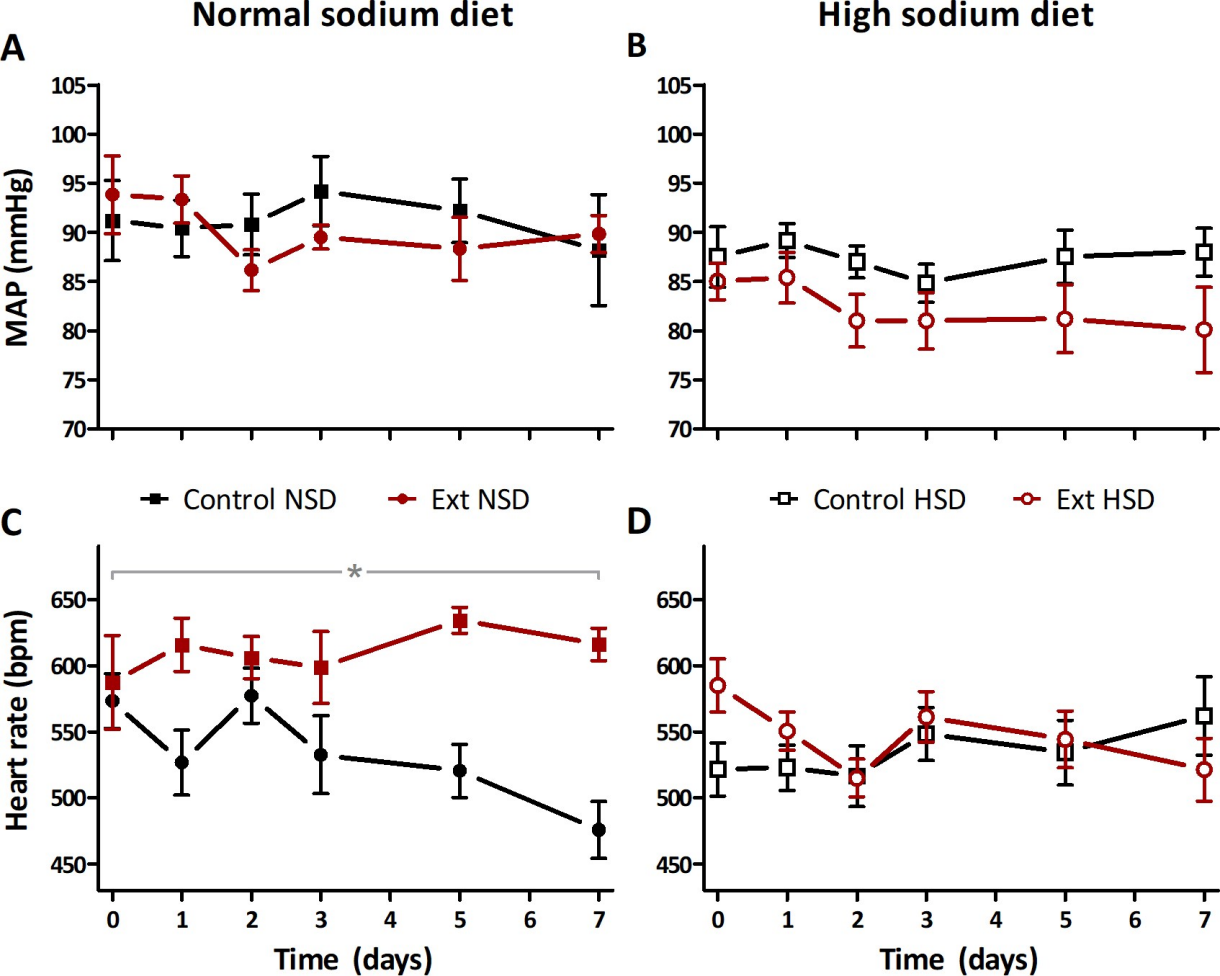
Ext1^+/-^Ext2^+/-^ mice have a normal MAP and are not salt sensitive. A) Ext1^+/-^Ext2^+/-^ (90±2 mmHg, n = 6) and control (91±2 mmHg, n = 5) mice had a comparable tail-cuff MAP on a NSD (p = 0.76). (B) Ext1^+/-^Ext2^+/-^ (82±2.0 mmHg, n = 10) and control mice (87±3 mmHg, n = 6, p = 0.15) had an equal MAP on a HSD. (C) On a NSD, heart rate was significantly higher in Ext1^+/-^Ext2^+/-^ mice than in controls (610±8 bpm vs. 534±8 bpm, p<0.001). (D) Heart rate was comparable in Ext1^+/-^Ext2^+/-^ and control mice on a HSD (546±11 bpm vs. 534±14 bpm, p = 0.51). *p<0.001. bpm, beats per minute; Ext, Ext1^+/-^Ext2^+/-^; HSD, high sodium diet; MAP, mean arterial pressure; NSD, normal sodium diet.

**Fig 4:**
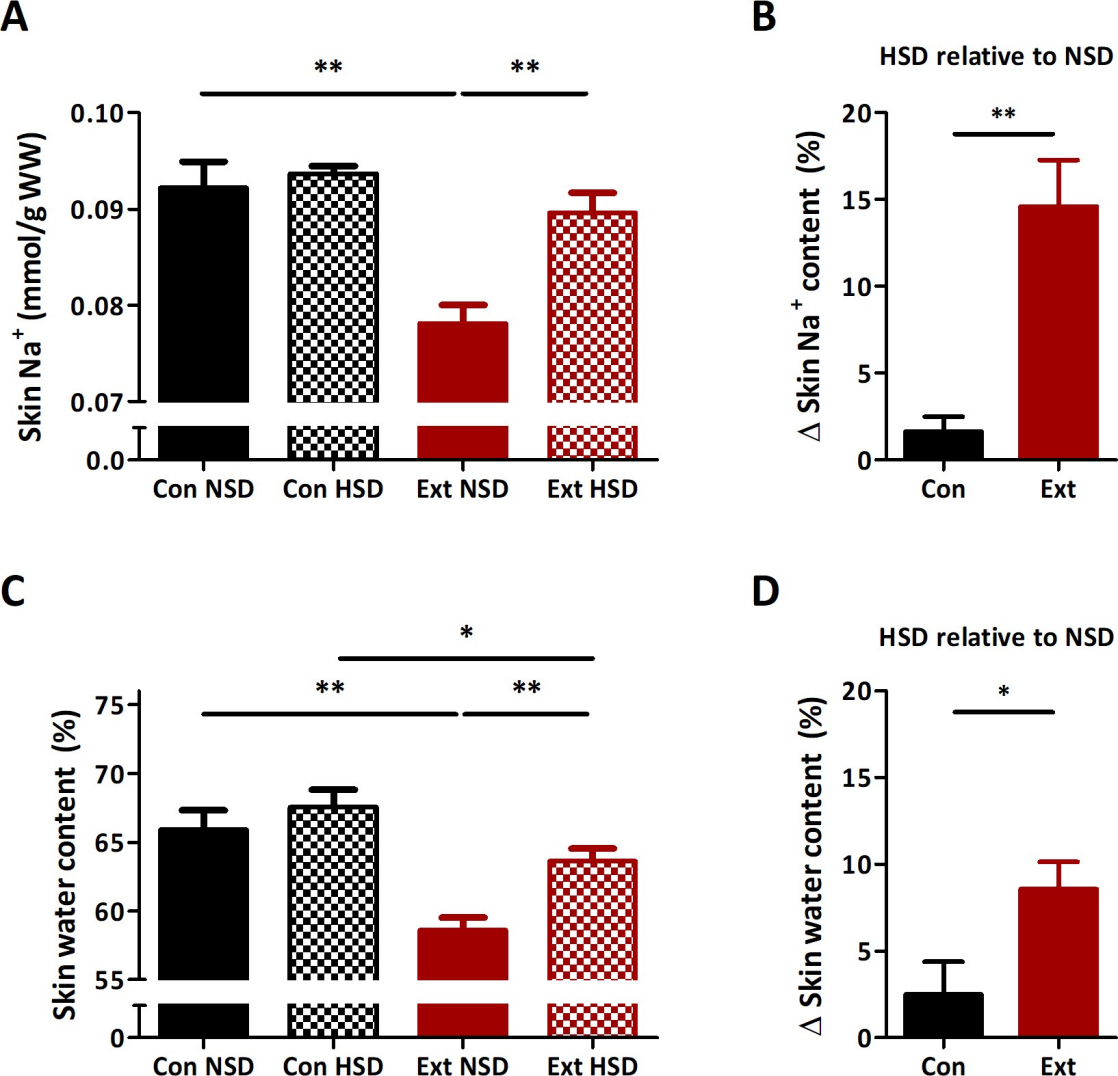
Ext1^+/-^Ext2^+/-^ mice have a decreased skin water and sodium content. (A) Skin sodium content was lower in Ext1^+/-^Ext2^+/-^ mice than in controls (p = 0.004). (A-B) HSD increased skin sodium content in Ext1^+/-^Ext2^+/-^ mice (15±6%, p = 0.003) to a level that were similar to controls on a HSD (p = 0.18). Skin sodium content was not affected by diet in controls (p = 0.43). (C) Skin water content of Ext1^+/-^Ext2^+/-^ mice was lower than in controls (p = 0.002). (C-D) After a HSD, skin water content increased (9±4%, p = 0.003) in Ext1^+/-^Ext2^+/-^ mice. Skin water content was not affected by diet in controls (p = 0.41). n = 5–6 for each condition. *p<0.05, **p<0.01. Con, controls; Ext, Ext1^+/-^Ext2^+/-^; HSD, high sodium diet; NSD, normal sodium diet.

To assess the consequences of impaired sodium storage capacity we assessed hemodynamics and osmoregulation. Baseline body weight was not different between both groups (Table 1). Mean arterial pressure (MAP) was similar in Ext1^+/-^Ext2^+/-^ and controls, but heart rate was significantly higher in Ext1^+/-^Ext2^+/-^ mice (Fig 3). We found a significantly higher plasma osmolality in Ext1^+/-^Ext2^+/-^ mice while plasma sodium concentration was found to be similar (Table 1). Ext1^+/-^Ext2^+/-^ mice had significant higher water intake than controls, whereas food intake was equal for both strains. Next, we assessed local sodium and water homeostasis in the skin compartment and found that skin sodium and water content were significantly decreased in Ext1^+/-^Ext2^+/-^ mice (Fig 4A–4C). Altogether, these data suggest that Ext1^+/-^Ext2^+/-^ mice were prone to volume depletion.

**Table 1:** Characteristics of Ext1^+/-^Ext2^+/-^ and control mice.

	NSD		HSD	
	Control	Ext	Control	Ext
**Plasma**	*(n = 11)*	*(n = 11)*	*(n = 5)*	*(n = 5)*
Na^+^ (mM)	150.8±2.1	149.9±1.5	155.6±0.5†	153.0±1.2†
K^+^ (mM)	5.4±0.3	5.4±0.5	4.4±0.5†	5.1±0.4
HCO3^-^ (mM)	17.4±3.8	18.0±2.7	18.3±1.8†	20.8±1.6
Glucose (mM)	9.0±1.6	9.1±1.9	7.5±1.6	7.3±0.5
Urea (mM)	7.9±0.8	8.6±0.5	6.0±0.4†	6.0±1.0†
Osmolality (mOsm/kg)	320±5	326±6*	323±5†	323±5
**Intake**	(n = 16)	(n = 13)	(n = 6)	(n = 11)
Water (ml/day)	3.9±0.1	4.7±0.1**	12.4±1.0‡	13.1±0.9‡
Food (g/day)	3.47±0.06	3.39±0.06	3.16±0.12	2.97±0.08
**Other**	(n = 22)	(n = 19)	(n = 11)	(n = 23)
Weight (g)	26.2±0.4	25.7±0.2	24.7±0.2†	23.1±0.3‡

Mean and standard error *p<0.05, **p<0.001 compared to control NSD (Independent samples Mann-Whitney U test or comparison of general linear model)

†p<0.05

‡p<0.001 compared to NSD (paired samples T-test).

Ext, Ext1^+/-^Ext2^+/-^, NSD, normal sodium diet; HSD, high sodium diet.

### Ext1^+/-^Ext2^+/-^ mice have an abnormal response to sodium excess

One week of high sodium intake did not affect MAP in control or Ext1^+/-^Ext2^+/-^ mice, but the increased heart rate of Ext1^+/-^Ext2^+/-^ mice at baseline normalized to values similar to controls (Fig 3). Fluid intake during high sodium intake was similar in both groups (Table 1). Skin sodium and water content in Ext1^+/-^Ext2^+/-^ mice normalized after high sodium intake while no changes were observed in controls (Fig 4). These data indicate that the initial volume depletion in Ext1^+/-^Ext2^+/-^ mice was corrected with high sodium intake.

To explain the absent hypothesized MAP increase in Ext1^+/-^Ext2^+/-^ mice we analysed whether a high sodium diet affected nonosmotic sodium storage capacity. Upon high sodium intake, skin heparan and dermatan sulfate content of Ext1^+/-^Ext2^+/-^ mice increased while skin GAG content remained unchanged in controls (Fig 1). High sodium intake also increased ESL thickness of control mice by 80% but the ESL of Ext1^+/-^Ext2^+/-^ mice remained undetectable (Fig 2).

**Fig 5:**
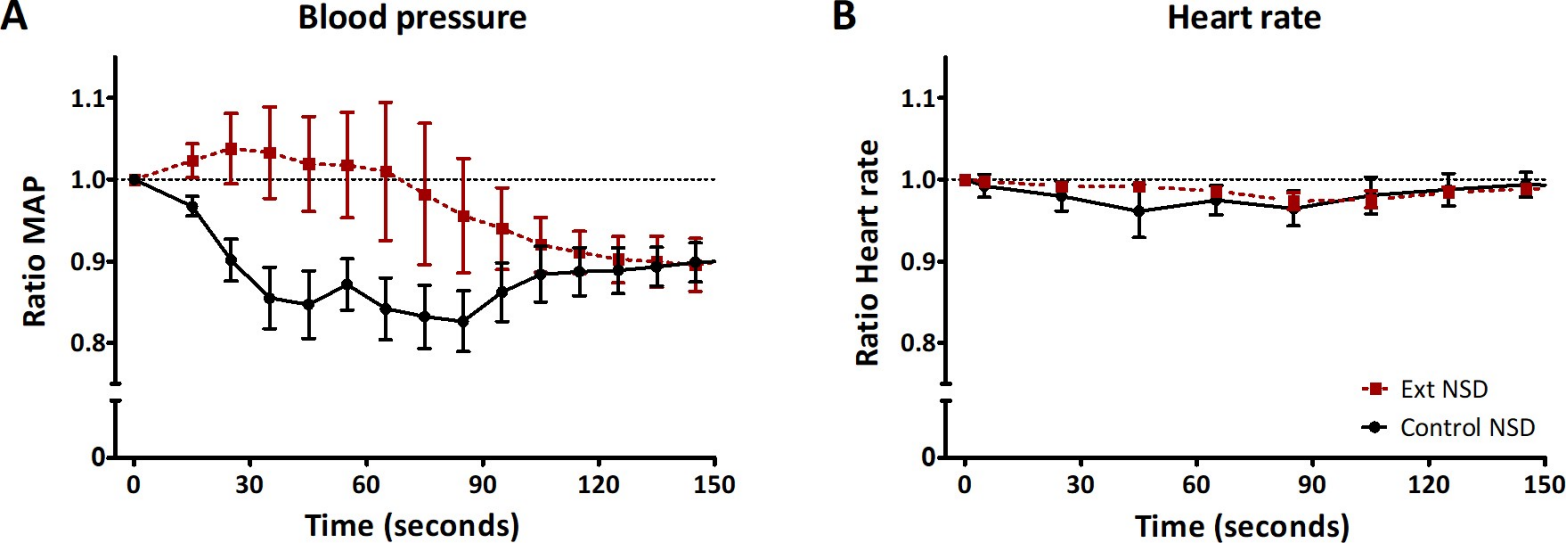
Ext1^+/-^Ext2^+/-^ mice show an abnormal MAP response after acute sodium loading. Control and Ext1^+/-^Ext2^+/-^ mice were subjected to rapid 1.8% NaCl infusion, which was finished at t = 0 seconds. (A) Ext1^+/-^Ext2^+/-^ (n = 5) and control mice (n = 6) showed a significant variation in intracarotid MAP response (Ext1^+/-^Ext2^+/-^ vs control, p = 0.02): control mice had a significant 15±4% (p = 0.02) decrease in MAP, while MAP of Ext1^+/-^Ext2^+/-^ mice did not change (3±6%, p = 0.65). At baseline, MAP was not different whereas heart rate of Ext1^+/-^Ext2^+/-^ mice was significantly higher (421±14 bpm) than in control mice (335±11 bpm, p = 0.005). (B) After infusion, we did not observe any change in heart rate in control (-2±2%) or Ext1^+/-^Ext2^+/-^ mice (-1±1%) (p = 0.49). Ext, Ext1^+/-^Ext2^+/-^; NSD, normal sodium diet.

To exclude potential obscuring effects of compensating mechanisms after a chronic sodium load that may affect MAP, such as changes in heart rate or sodium storage capacity, we investigated an acute sodium load, which showed a distinct response in Ext1^+/-^Ext2^+/-^ and control mice. Following an acute hypertonic sodium load, 6 out of 6 controls showed a significant physiologic decrease in MAP (18±4 mmHg, p = 0.006) that was not observed in Ext1^+/-^Ext2^+/-^ mice, of which 4 out of 5 showed a MAP increase that was on average 6±3 mmHg (p = 0.16)(Fig 5A). This effect was transient and after 2 minutes MAP was similar in both groups. This distinct MAP response was not accompanied by any change in heart rate in Ext1^+/-^Ext2^+/-^ or control mice (Fig 5B). MAP and heart rate remained similar to baseline values during the next ten minutes.

### Ext1^+/-^Ext2^+/-^ mice are characterized by endothelial dysfunction

Finally, we analysed whether defective heparan sulfate polymerization affected BP via impaired endothelial function, which may be anticipated as a result of ESL damage. We assessed *ex-vivo* vasodilation with methacholine dose-response curves. We observed that Ext1^+/-^Ext2^+/-^ mice had a significantly impaired ability for maximum vasodilation (35±10%, n = 5) in comparison to controls (59±4%, n = 6; p = 0.02). High sodium intake did not affect vasodilation in Ext1^+/-^Ext2^+/-^ mice (42±5%, n = 7, p = 0.21) but resulted in a non-significant decrease of vasodilation ability in control mice (43±11%, n = 5, p = 0.10) to a level that was similar to Ext1^+/-^Ext2^+/-^ mice (S2 Fig).

## Discussion

In this study, we examined the effects of defective heparan sulfate GAG polymerization on sodium and water homeostasis. Our study demonstrates that Ext1^+/-^Ext2^+/-^ mice have an impaired sodium storage capacity in the skin and ESL and display an abnormal sodium and water balance indicating that heparan sulfate GAGs are crucial for normal sodium and water homeostasis.

Heterozygous knock-out of Ext1 and Ext2 significantly affected sodium storage capacity, both in the skin and the ESL. Although skin heparan sulfate content was similar in Ext1^+/-^Ext2^+/-^ and control mice, the skin sodium-to-heparan sulfate ratio was significantly lower in Ext1^+/-^Ext2^+/-^ mice suggesting that the sodium-storing function of heparan sulfates is impaired by defective polymerization. Also, ESL thickness was reduced with 77% in Ext1^+/-^Ext2^+/-^ mice indicating that heparan sulfate polymerization is crucial for normal ESL dimensions. These data are in accordance with a previous study that assessed the effects of heterozygous knock-out of Ext1 or Ext2 on ESL thickness.[20]

The reduced capacity for local sodium storage was associated with abnormal sodium and water homeostasis. On a normal diet Ext1^+/-^Ext2^+/-^ mice had a higher heart rate, increased fluid intake and decreased skin sodium and water content, indicating volume depletion. The decreased capacity for local sodium storage due to dysfunctional heparan sulfates may lead to lower skin sodium and water content but cannot explain loss of sodium and water. In this respect, sodium and water may be lost via the kidney or skin as a result of an impaired renal concentrating mechanism or impaired skin electrolyte gradient formation, which were restored by a high sodium diet.[11, 12, 28] These hypotheses were not investigated in the current study but the potential role of renal sodium and water loss is supported by recent studies that demonstrated that heparan sulfate GAGs are abundantly present in the renal medulla and show increased sulfation rates after a high sodium diet.[29, 30] Altogether, these data suggest that defective heparan sulfate polymerization results in loss of sodium and water.

We hypothesized that Ext1^+/-^Ext2^+/-^ mice would show a sodium-induced BP increase because of the impaired local sodium storage capacity. However, we found that only an acute sodium load resulted in a different BP response in Ext1^+/-^Ext2^+/-^ mice. The absent BP effect in Ext1^+/-^Ext2^+/-^ after a high sodium diet may be explained by the volume depletion present at baseline. The normalization of heart rate and skin sodium content after high sodium intake indicates a certain degree of volume expansion after high sodium intake, without BP effects. The distinct effects of acute and chronic sodium loading on BP can be explained by the compensatory decrease in heart rate and increase in skin GAG content that were observed after high sodium intake but may not affect the hemodynamic effects of an acute sodium load. A potential limitation of this study is the use of tail-cuff BP measurements. Although previous studies have shown that tail-cuff measurements are accurate and able to detect blood pressure differences, such measurements are inferior to telemetry and may result in false-negative results in small sample sizes.[31, 32]

The different BP response of Ext1^+/-^Ext2^+/-^ mice after acute sodium loading may be explained by the reduced local sodium storage capacity in the skin and ESL. This reduction impairs the ability to instantaneously bind and osmotically inactivate the infused sodium, thereby increasing BP. Because an intact ESL is also essential for shear-mediated nitric oxide production, we examined endothelial function in Ext1^+/-^Ext2^+/^ mice. We found an impaired vasodilation response that may contribute to the distinct BP response after acute hypertonic saline infusion. This is supported by previous studies that have demonstrated that the transient BP reduction, which is normally seen after rapid hypertonic NaCl infusion and was present in controls, but absent in Ext1^+/-^Ext2^+/-^ mice, is caused by a decrease in vascular resistance [33–36] This transient BP reduction could not be prevented by hexamethonium, a nicotinic cholinergic antagonist, indicating that the decrease in vascular resistance is not of higher neurological origin but is caused by local factors that influence vasodilation such as nitric oxide.[33, 34]

In control mice, high sodium intake increased ESL thickness suggesting that ESL volume is affected by sodium. The observed increase in ESL thickness may represent a first defence mechanism to prevent a sodium-mediated BP increase by reducing the amount of circulating osmotically active sodium. A similar defence mechanism is observed in aquatic species that increase their extracellular GAG quantity and sulfation with the salinity of their environment.[37] This hypothesis is also in line with data from diabetic and chronic kidney disease patients, which have a perturbed ESL and are prone to develop hypertension and volume expansion.[38, 39] Likewise, ESL restoration with sulodexide, a highly purified mixture of GAGs, has been shown to lower BP.[40, 41] This may either be attributed to an increase in nonosmotic sodium storage capacity, nitric oxide bioavailability or both.[42]

Considering the anatomic location, local sodium storage by skin and ESL heparan sulfates may differently affect sodium and water homeostasis. An intact ESL may help to prevent acute sodium excess and preserve endothelial function on the short term. On the other hand, sodium accumulation in the skin has been associated with conditions of sodium overload such as heart failure, dialysis, hypertension and hyperaldosteronism, indicating that skin sodium accumulation, which may seem a beneficial compensatory mechanism on first sight, is a sign of severe sodium excess with potential negative effects.[43–46] Hypersalinity, for example, is associated with a reduced vasodilatory response and may thereby affect BP.[47–49]

In conclusion, this study demonstrates that heparan sulfate GAGs play a crucial role in sodium and water homeostasis. Future research with targeted knock-out of endothelial and skin heparan sulfate GAGs are needed to determine whether sodium storage in the skin and ESL have differential effects on sodium and water balance. Investigation of this interaction may help to understand the pathophysiology of altered sodium and water homeostasis in common diseases such as diabetes mellitus and chronic kidney disease, which are characterized by altered sodium and water homeostasis, high skin sodium content and lower ESL-mediated sodium storage capacity.

## Supporting information

S1 Fig Intravital microscopy data analysis. (PDF)

S2 Fig Wire myography data. (PDF)
