# Supplementary material for: Abnormal sodium and water homeostasis in mice with defective heparan sulfate polymerization
Source: PLoS One. 2019 Jul 31;14(7):e0220333. doi: 10.1371/journal.pone.0220333 (PMC6668793; doi:10.1371/journal.pone.0220333)
Supplement: S1 Fig — (PDF) [file pone.0220333.s001.pdf]

S1 Figure. Intravital microscopy data analysis.

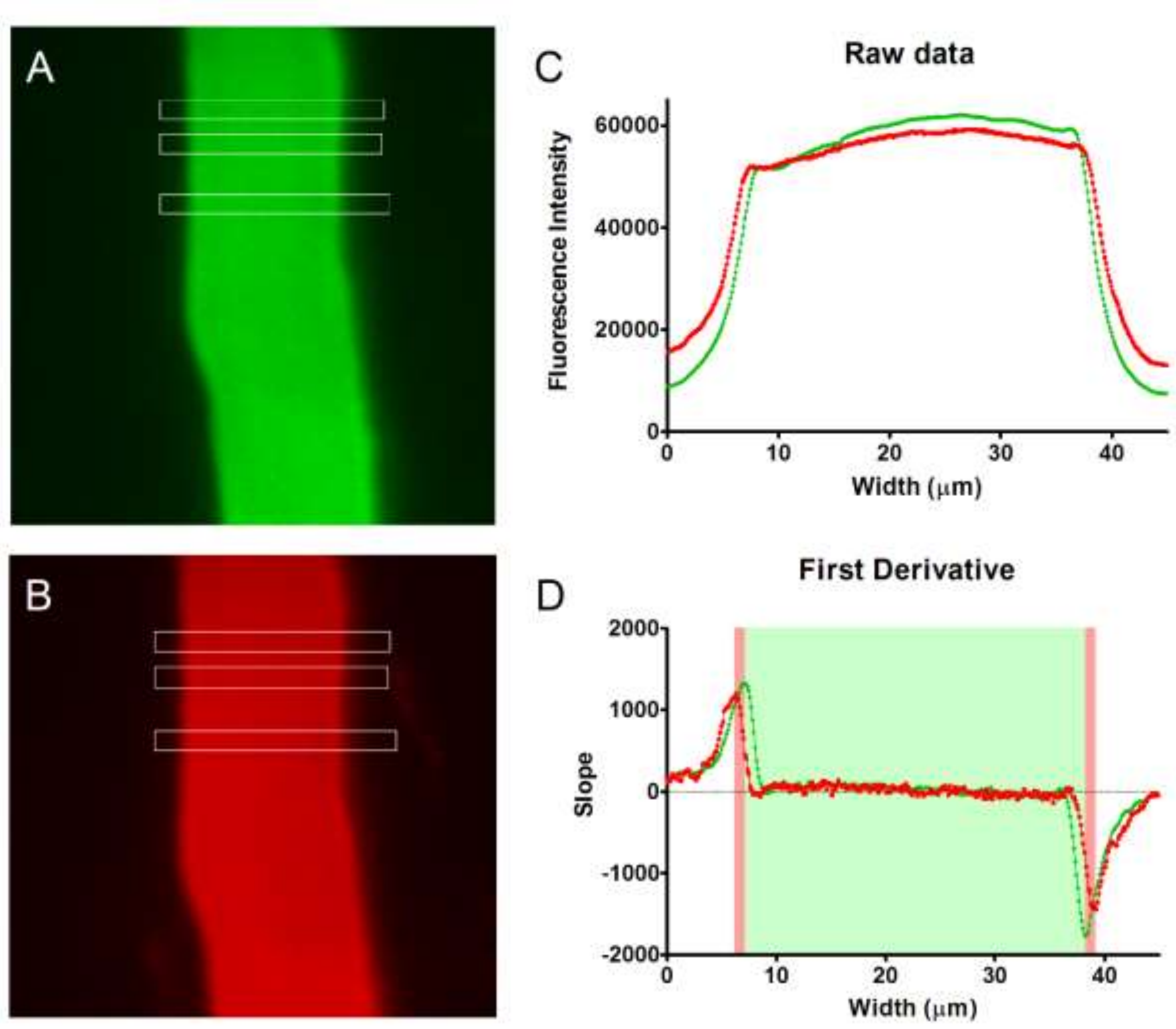

Examples of images of FITC-Dx500 (A) and TRITC-Dx40 columns (B) in which we drew three small rectangles to analyse fluorescence intensity (C). We calculated the inflection points (maximal value of the first derivative (D)), to determine the edge of fluorescent columns and calculate the endothelial surface layers thickness by subtracting the fluorescent column of the FITC-Dx500 from the TRITC-Dx40 column (vertical red bars). Dx500, dextran 500 kDa; Dx40, dextran 40 kDa.
