# Supplementary material for: Abnormal sodium and water homeostasis in mice with defective heparan sulfate polymerization
Source: PLoS One. 2019 Jul 31;14(7):e0220333. doi: 10.1371/journal.pone.0220333 (PMC6668793; doi:10.1371/journal.pone.0220333)
Supplement: S2 Fig — (PDF) [file pone.0220333.s002.pdf]

S2 Figure. Vasodilation is impaired in Ext1$^{+/-}$Ext2$^{+/-}$ mice.

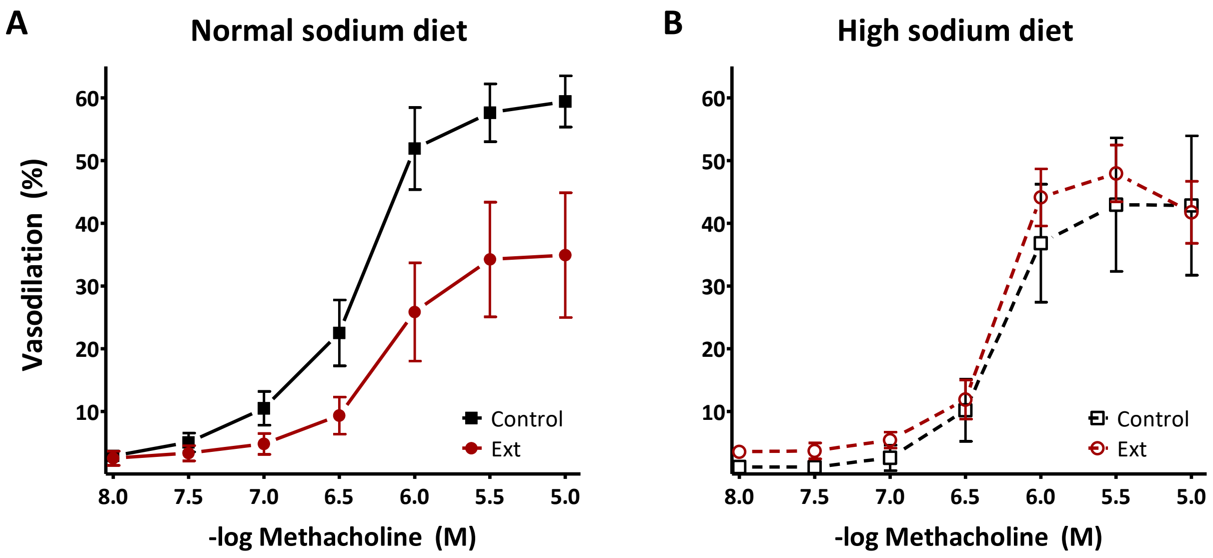

Ex-vivo vasodilation of mesenteric arteries was assessed with wire myography at increasing methacholine concentrations after a normal and high salt diet. (A) Vasodilation in Ext1$^{+/-}$Ext2$^{+/-}$ mice (Ext, 35±10%, n=5) was significantly impaired when compared to control mice (59±4%, n=6) during a normal sodium diet (p=0.02). (B) Vasodilation responses of Ext1$^{+/-}$Ext2$^{+/-}$ (42±5%, n=7) and control mice (43±11%, n=5) were equal during high sodium intake (p=0.62).
